# Isothiocyanates Potentiate Tazemetostat-Induced Apoptosis by Modulating the Expression of Apoptotic Genes, Members of Polycomb Repressive Complex 2, and Levels of Tri-Methylating Lysine 27 at Histone 3 in Human Malignant Melanoma Cells

**DOI:** 10.3390/ijms25052745

**Published:** 2024-02-27

**Authors:** Ioannis Anestopoulos, Ioannis Paraskevaidis, Sotiris Kyriakou, Lambrini E. Giova, Dimitrios T. Trafalis, Sotiris Botaitis, Rodrigo Franco, Aglaia Pappa, Mihalis I. Panayiotidis

**Affiliations:** 1 Department of Cancer Genetics, Therapeutics & Ultrastructural Pathology, The Cyprus Institute of Neurology & Genetics, 6 Iroon Avenue, Ayios Dometios, Nicosia 2371, Cyprus; ioannisa@cing.ac.cy (I.A.); yiannisparaskevaidis@gmail.com (I.P.); sotirisk@cing.ac.cy (S.K.); lamprini.giova@gmail.com (L.E.G.); 2 Laboratory of Pharmacology, Medical School, National & Kapodistrian University of Athens, 11527 Athens, Greece; dtrafal@med.uoa.gr; 3 Department of Surgery, School of Medicine, University Hospital, Democritus University of Thrace, 68100 Alexandroupolis, Greece; smpotait@med.duth.gr; 4 School of Veterinary Medicine & Biomedical Sciences, University of Nebraska-Lincoln, Lincoln, NE 68583, USA; rodrigo.franco@unl.edu; 5 Redox Biology Centre, University of Nebraska-Lincoln, Lincoln, NE 68583, USA; 6 Department of Molecular Biology & Genetics, Democritus University of Thrace, 68100 Alexandroupolis, Greece; apappa@mbg.duth.gr

**Keywords:** melanoma, Tazemetostat, isothiocyanates, epigenetics, therapeutics, apoptosis

## Abstract

In this study, we utilized an in vitro model consisting of human malignant melanoma as well as non-tumorigenic immortalized keratinocyte cells with the aim of characterizing the therapeutic effectiveness of the clinical epigenetic drug Tazemetostat alone or in combination with various isothiocyanates. In doing so, we assessed markers of cell viability, apoptotic induction, and expression levels of key proteins capable of mediating the therapeutic response. Our data indicated, for the first time, that Tazemetostat caused a significant decrease in viability levels of malignant melanoma cells in a dose- and time-dependent manner via the induction of apoptosis, while non-malignant keratinocytes were more resistant. Moreover, combinatorial treatment protocols caused a further decrease in cell viability, together with higher apoptotic rates. In addition, a significant reduction in the Polycomb Repressive Complex 2 (PRC2) members [e.g., Enhancer of Zeste Homologue 2 (EZH2), Embryonic Ectoderm Development (EED), and suppressor of zeste 12 (SUZ12)] and tri-methylating lysine 27 at Histone 3 (H3K27me3) protein expression levels was observed, at least partially, under specific combinatorial exposure conditions. Reactivation of major apoptotic gene targets was determined at much higher levels in combinatorial treatment protocols than Tazemetostat alone, known to be involved in the induction of intrinsic and extrinsic apoptosis. Overall, we developed an optimized experimental therapeutic platform aiming to ensure the therapeutic effectiveness of Tazemetostat in malignant melanoma while at the same time minimizing toxicity against neighboring non-tumorigenic keratinocyte cells.

## 1. Introduction

Malignant melanoma is characterized by high incidence rates and is the deadliest type among all types of skin cancer, especially in its metastatic form [1,2,3]. Although, in recent years, a number of therapeutic agents have been developed, the overall 5-year survival rates are still considered low, especially in patients with advanced and/or metastatic types of the disease. This, in turn, highlights the urgent need for the development of more efficient and safer therapeutic options [4]. On the other hand, there is increasing evidence that melanoma pathogenesis is also associated with specific aberrant epigenetic modifications, leading to drug-acquired resistance, evasion of the immune system, apoptosis, and increased metastatic potential. Thus, the reversal of an abnormal epigenetic landscape appears to be a promising therapeutic approach, such as in the case of EZH2 (Enhancer of Zeste Homologue 2; a histone methyltransferase) over-expression and its role in melanoma development and progression [5,6,7,8,9].

EZH2 is a member of the Polycomb Group (PcG) of proteins that form two major Polycomb Repressive Complexes, namely PRC1 (Polycomb Repressive Complex 1) and PRC2 (Polycomb Repressive Complex 2). Moreover, EZH2 serves as the catalytic component of the PRC2 complex, which modulates gene expression via its intrinsic histone methyltransferase activity, ability to recruit DNA methyltransferases (DNMTs), and interaction with histone deacetylases (HDACs). Consequently, EZH2 is responsible for tri-methylating lysine (K)-27 residues at histone H3 (H3K27me3) known to be associated with gene repression. However, EZH2 can also interact with the other two members of the PRC2 complex [Embryonic Ectoderm Development (EED) and suppressor of zeste 12 (SUZ12)] in order to be fully functional [10]. As a result, EZH2 has been shown to regulate the progression of melanoma by negatively regulating a number of tumor suppressor genes (TSGs) implicated in cell transformation and proliferation, evasion of senescence and apoptosis, as well as increased invasiveness and metastasis. In this context, EZH2 has emerged as an important target for the development of various inhibitors, including Tazemetostat (TAZ; Tazveric, EPZ-6438), a first-in-class potent and selective EZH2 inhibitor used in the treatment of advanced epithelioid sarcoma [11]. 

Isothiocyanates (ITCs) are the bioactive components of cruciferous vegetables obtained via hydrolysis of their biologically inert precursors known as glucosinolates (GLs). There are different major types, including sulforaphane (SFN), iberin (IBN), allyl (AITC), benzyl (BITC), and phenethyl (PEITC) isothiocyanates, all of which have been reported to exert considerable anti-cancer activity via the induction of cell cycle arrest and apoptosis as well as inhibition of angiogenesis and metastasis, etc. [12,13,14]. In addition, BITC, PEITC, SFN, IBN, and AITC have been shown to induce significant cytotoxicity in malignant melanoma cells by modulating distinct aberrant epigenetic pathways, including, at least in part, methylation and acetylation marks of specific lysine residues at histones H3 and H4 via the modulation of various histone acetyltransferases (HATs), deacetylases (HDACs) and methyltransferases (HMTs) [15,16,17].

**Figure 1:**
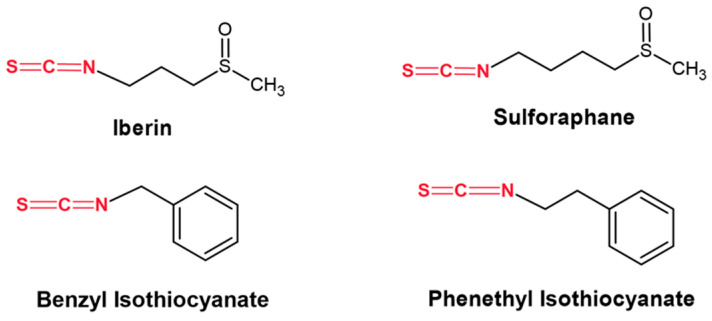
The structure of iberin, sulforaphane, benzyl, and phenethyl isothiocyanates used in the current study. The isothiocyanate moiety is shown in red.

In the current study, our aim was to investigate the underlying mechanism(s) by which the therapeutic effectiveness of Tazemetostat in malignant melanoma cells is induced while, at the same time, minimal cytotoxicity against neighboring non-tumorigenic keratinocyte cells is ensured in combinatorial treatment protocols utilizing various ITCs (Figure 1). Specifically, our objective was two-fold: (i) to characterize the anticancer effect of the epigenetic drug TAZ as a single agent and (ii) to evaluate its anticancer potency in combinatorial treatment protocols with SFN, IBN, PEITC, and BITC against an experimental model of human malignant melanoma. In this context, we aimed to characterize the underlying molecular mechanism(s) of enhanced apoptotic induction and the overall involvement of the main members of the PRC2 complex in this model of malignant melanoma.

## 2. Results

### 2.1. Optimization of the Experimental Exposure Model

**Figure 2:**
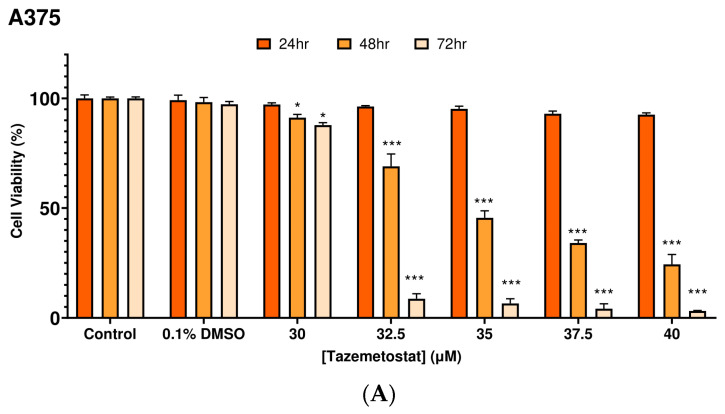

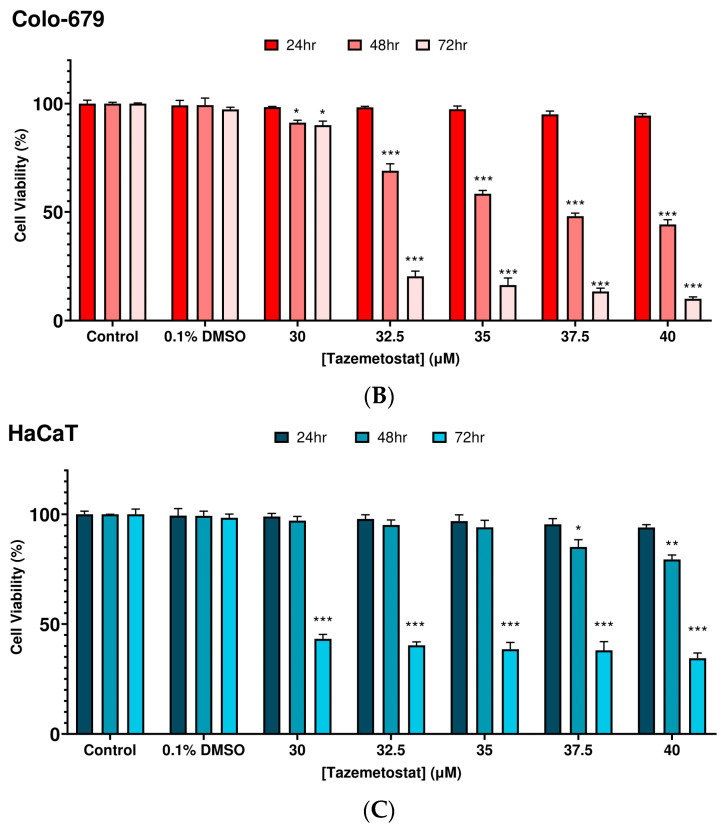
Cytotoxic profile of TAZ against malignant melanoma and non-tumorigenic immortalized keratinocyte cells. A375 (**A**), Colo-679 (**B**), and HaCaT (**C**) cells were subjected to increasing concentrations of TAZ for 24–72 h. Cell viability levels were determined by using the Alamar Blue assay. All data are expressed as means of five replicates ± standard error of the mean (SEM) and are representative of three independent experiments. Statistical significance is indicated by * *p* < 0.05, ** *p* < 0.01, *** *p* < 0.001 relative to corresponding 0.1% DMSO controls.

All cell lines were exposed to a range of TAZ concentrations (0–40 μM) over 24–72 h of exposure. TAZ induced a cytotoxicity as early as 48 h of exposure in A375 cells, an effect observed at the highest range of concentrations (35–40 μM). Indeed, a 50–75% decline in A375 cell viability levels was observed, which was further substantiated over 72 h of exposure, reaching an overall viability of about 10% (Figure 2A). On the other hand, Colo-679 cells appeared to be more resistant when compared to A375, as a slight decline in cell viability levels was observed (10–15%) at 24–48 h of exposure at all TAZ concentrations. However, a more profound cytotoxic effect was observed at 72 h of exposure, causing a reduction in cell viability to about 60–65% (Figure 2B). Finally, TAZ was shown to exert a safe cytotoxic profile against HaCaT cells, as viability levels were either unaffected or minimally reduced (10–15%) after 24–48 h, while cytotoxicity was observed only at 72 h of exposure (Figure 2C). Overall, EC_50_ values of TAZ were determined to be 35.0 and 37.5 μM in A375 and Colo-679 melanoma cells for 48 h of exposure, respectively, while no EC_50_ values were determined for any other time point of exposure. Next, we determined the combinational cytotoxic effect of TAZ together with each of SFN, IBN, PEITC, and BITC. Based on our previous works, exposure to 10 μM of any of these ITCs exhibited significant cytotoxicity at 48 h against various melanoma cell lines [15,16]. Consequently, we developed an optimized protocol of combinatorial exposures consisting of the following conditions: (i) TAZ (35 μM)/SFN (10 μM); (ii) TAZ (15 μM)/IBN (7.5 μM); (iii) TAZ (35 μM)/PEITC (10 μM); and (iv) TAZ (15 μM)/BITC (7.5 μM).

**Figure 3:**
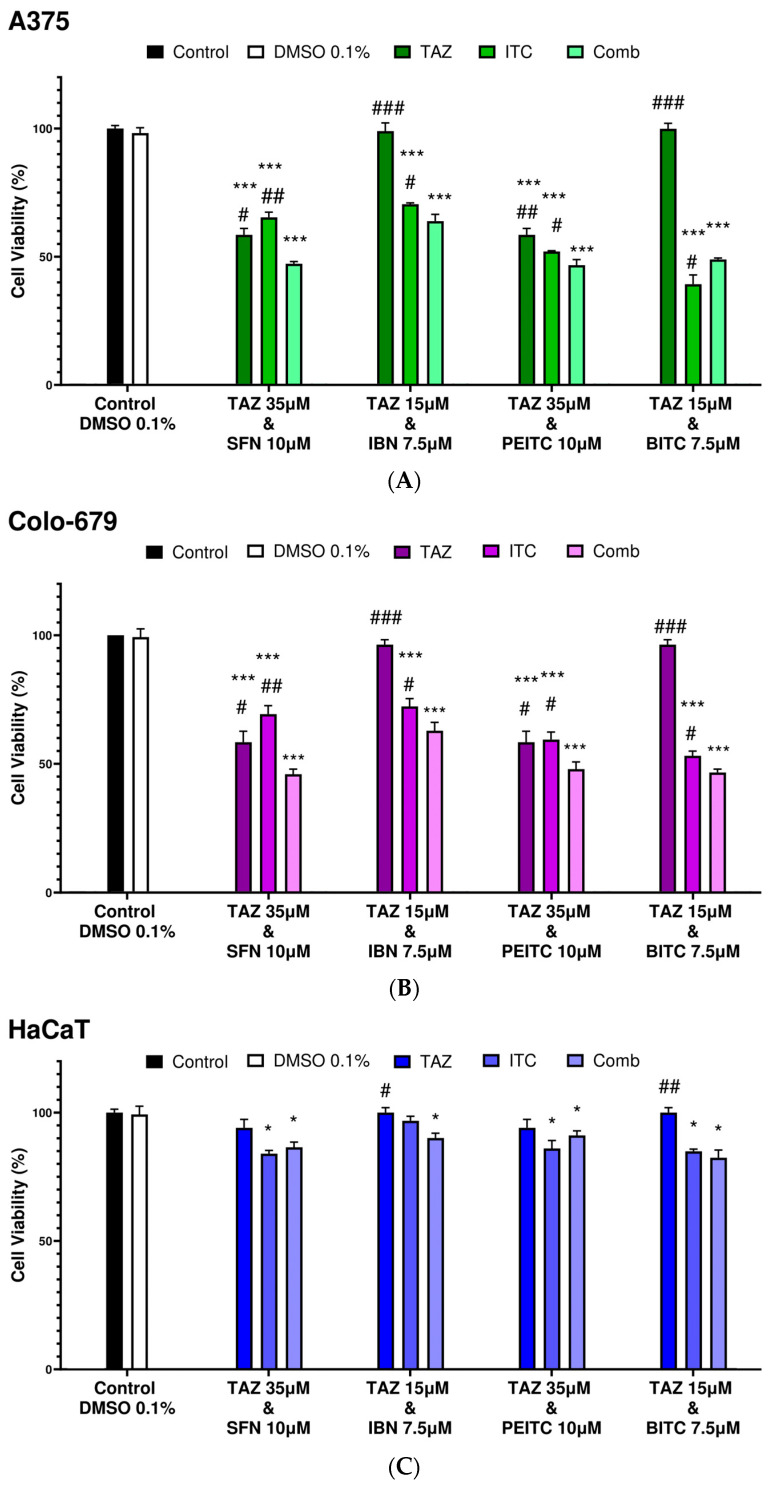
Cytotoxic profile of either single or combinatorial exposures of TAZ with each ITC against malignant melanoma and non-tumorigenic immortalized keratinocyte cells. A375 (**A**), Colo-679 (**B**), and HaCaT (**C**) cells were subjected to TAZ and/or each ITC alone or combined. Cell viability levels were determined using the Alamar Blue assay. All data are expressed as means of five replicates ± SEM and are representative of three independent experiments. Statistical significance is indicated by * *p* < 0.05, and *** *p* < 0.001 relative to corresponding 0.1% DMSO controls and # *p* < 0.05, ## *p* < 0.01, and ### *p* < 0.001 relative to the corresponding combinatorial exposure.

In our next series of experiments, in A375 cells, combined exposure of TAZ with each ITC resulted in the potentiation of cytotoxicity as opposed to exposure with each agent alone. Specifically, the co-exposure of TAZ (35 μM) with either SFN or PEITC slightly enhanced cytotoxicity, while the co-exposure of TAZ (15 μM) with either IBN or BITC potentiated cytotoxicity by about 35% and 50%, respectively (Figure 3A). On the other hand, a similar cytotoxic effect was observed in Colo-679 cells, where the co-exposure of TAZ (35 μM) with each ITC enhanced cytotoxicity to a variable degree. In particular, co-exposure with either SFN or PEITC enhanced cytotoxicity by about 15% in both cases, whereas the co-exposure of TAZ (15 μM) with either IBN or BITC further potentiated cytotoxicity by about 35% and 55%, respectively (Figure 3B). Finally, the co-exposure of any TAZ concentration with each ITC exerted limited cytotoxicity against HaCaT cells, indicating their resistance to co-exposures (Figure 3C).

### 2.2. Combination Index Analyses for Profiling Co-Exposure Interactions

Next, we sought to determine the nature of interaction between TAZ with each ITC in A375 cells by means of combination index (CI) analysis (Table 1). The isobolographic analysis indicated that the interactions between each ITC and TAZ were either synergistic (CI < 1) or additive (CI = 1) according to different experimental conditions. Specifically, the co-exposure of TAZ with SFN or PEITC exerted a synergistic action since CIs were 0.89 and 0.79, respectively, whereas when TAZ was combined with either IBN or BITC, an additive action was observed (CIs: 1.02 and 1.09, respectively) (Figures S3 and S4).

### 2.3. Caspase-3 Activity Levels as an Index of Apoptotic Induction

**Figure 4:**
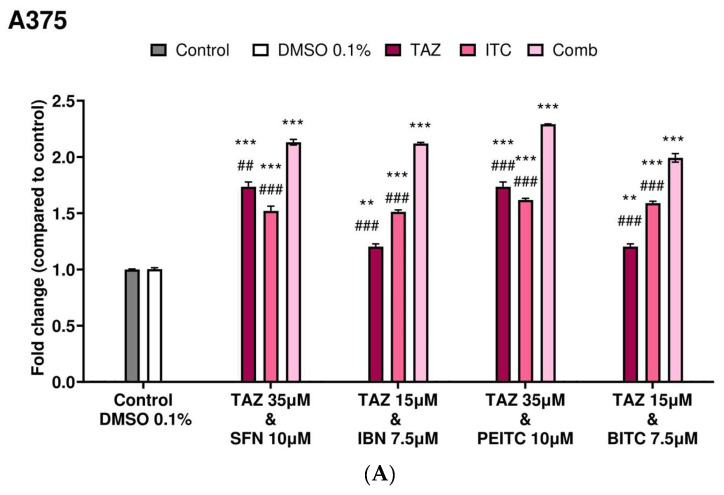

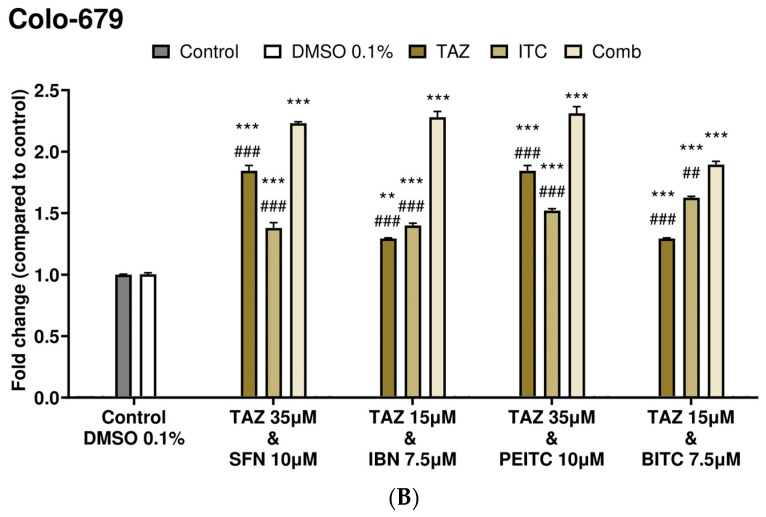
Determination of caspase-3 activity levels in malignant melanoma cells. A375 (**A**) and Colo-679 (**B**) cells were subjected to either TAZ or each ITC alone or in combination for 48 h. Caspase-3 activity levels were estimated by a commercial fluorimetric multiplex assay kit. All data are expressed as means of five replicates ±SEM and are representative of three independent experiments. Statistical significance is indicated by ** *p* < 0.01, and *** *p* < 0.001 relative to corresponding 0.1% DMSO controls and ## *p* < 0.01, and ### *p* < 0.001 relative to the corresponding combinatorial exposure.

Overall, caspase-3 activity was significantly increased in both A375 and Colo-679 cells exposed to the highest (35 μM) as opposed to the lower (15 μM) TAZ concentration when compared to non-exposed (control) cells. Moreover, a significant induction in caspase-3 activity was also observed in all exposures to each ITC as single agents. However, co-exposures of TAZ with each ITC further potentiated apoptotic induction in A375 cells (Figure 4A). A similar pattern was also observed in Colo-679 cells as, once again, the co-exposures of TAZ with each ITC potentiated apoptosis to levels comparable to those observed in A375 cells (Figure 4B). 

### 2.4. Differential Gene Expression Response of Major Apoptotic Pathways

**Figure 5:**
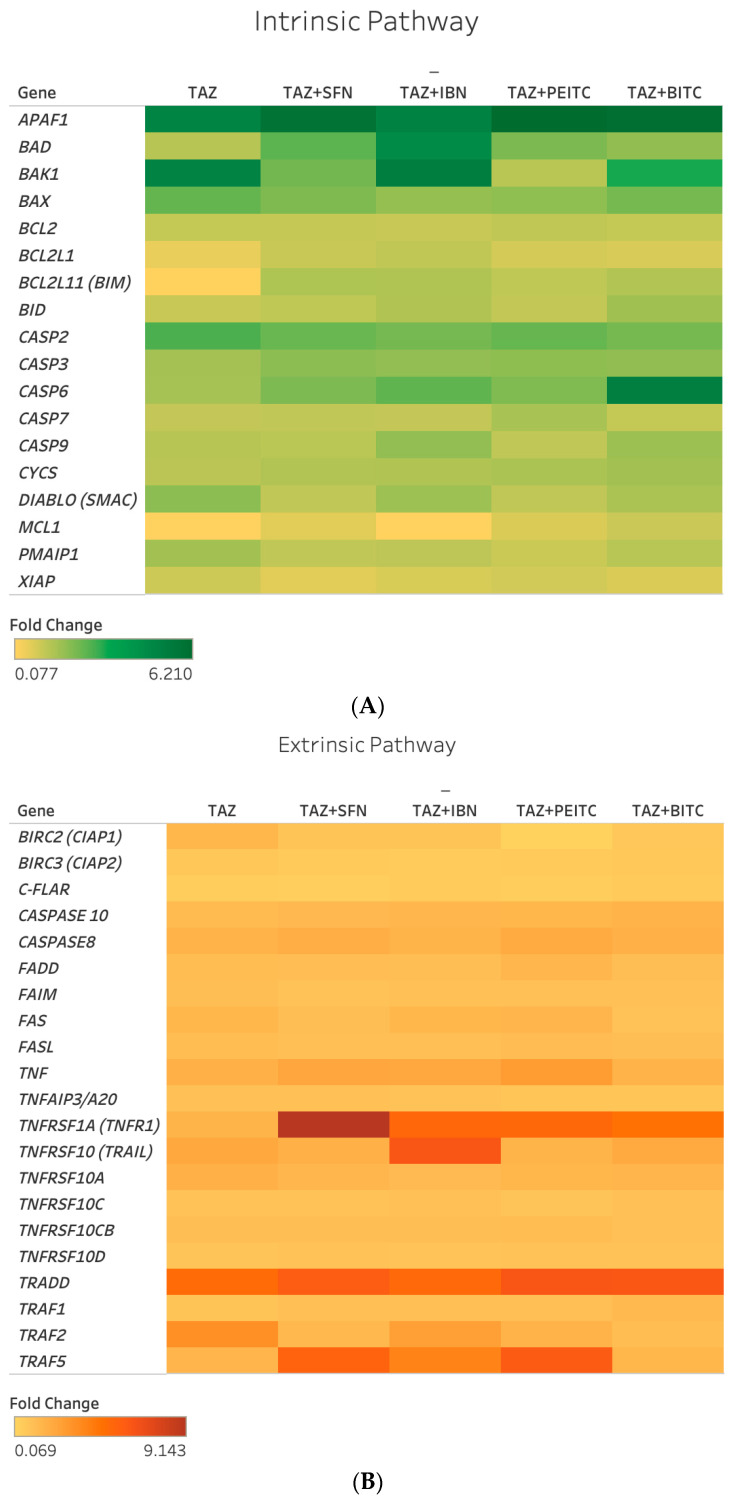
Heat map representation of expression levels of genes involved in intrinsic (**A**) and extrinsic (**B**) in A375 cells exposed to TAZ as a single agent and/or in combined exposures with each ITC. Fold changes in gene expression levels were determined by real-time PCR (RT-PCR) and are representative of three independent experiments. Gene expression data were normalized to *β-actin*, using the 2^−ΔΔCt^ method, and were expressed as fold change compared to untreated (control) samples. Different exposure conditions are represented in the x-axis, while genes are represented in the y-axis. Color saturation represents the magnitude of gene expression.

**Figure 6:**
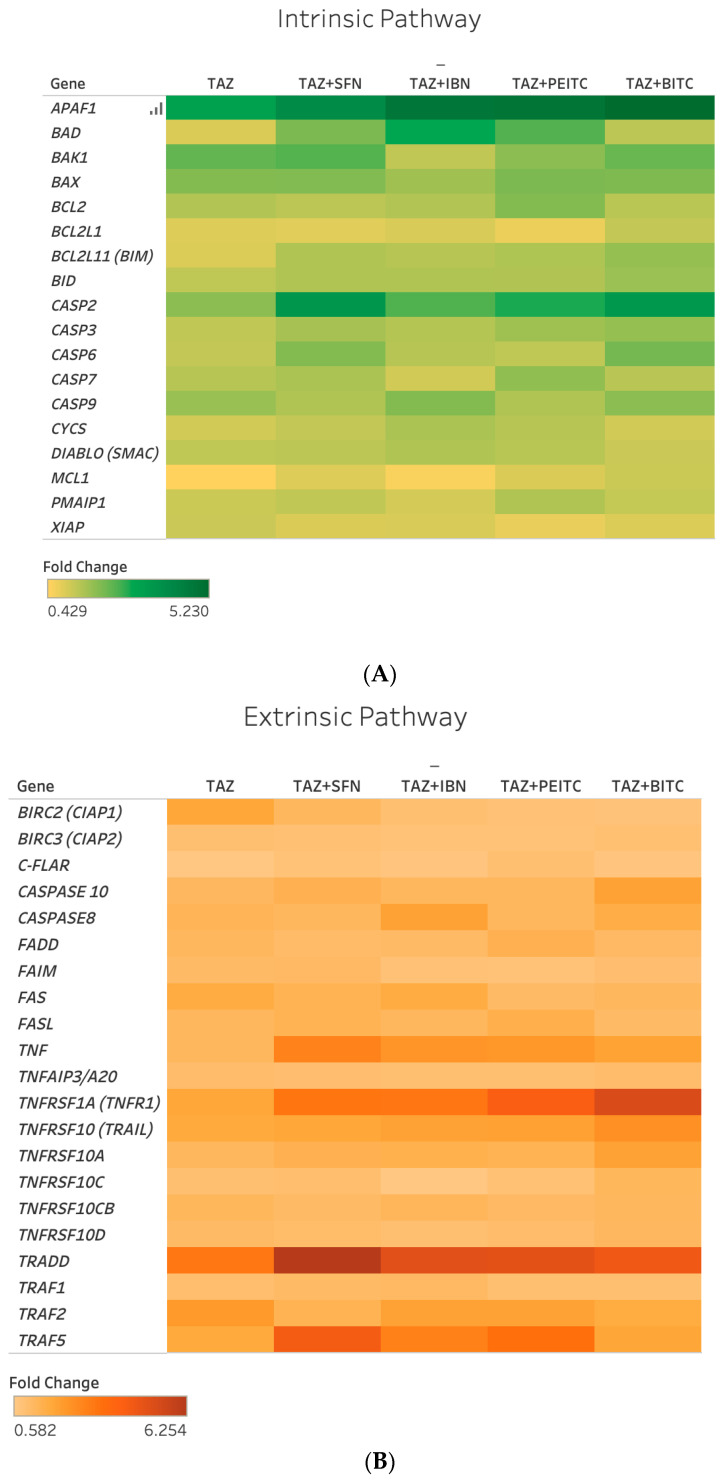
Heat map representation of expression levels of genes involved in intrinsic (**A**) and extrinsic (**B**) in Colo-679 cells exposed to TAZ as a single agent and/or in combined exposures with each ITC. Fold changes in gene expression levels were determined by real-time PCR (RT-PCR) and are representative of three independent experiments. Gene expression data were normalized to *β-actin*, using the 2^−ΔΔCt^ method, and were expressed as fold change compared to untreated (control) samples. Different exposure conditions are represented in the x-axis, while genes are represented in the y-axis. Color saturation represents the magnitude of gene expression.

Next, we evaluated, by RT-PCR, the effect of TAZ in regulating the expression of genes implicated in intrinsic and extrinsic apoptotic as well as anti-apoptotic pathways. Selection criteria included a higher than 2-fold regulation (up or down) in the majority of co-exposure conditions in both A375 and Colo-679 cells (Figure 5 and Figure 6).

Overall, a number of intrinsic (*BAD*, *BAK1*, and *APAF1*), extrinsic (*TNF*, *TNFRSF1A*, *TRADD*, and *TRAF5*), and other (*CASP2*) apoptotic genes were found to be upregulated following different co-exposure conditions in both cell lines (Tables S2 and S3). In addition, *CASP6* was upregulated, while *c-FLAIR* and *BIRC3* were downregulated but only in A375 cells. Interestingly, in the cases of *BAD*, *APAF1*, *TNF*, *TNFRSF1A*, *TRADD*, and *TRAF5*, the majority of co-exposures potentiated TAZ alone expression levels to a variable degree in both cell lines (Tables S2 and S3).

### 2.5. Effect of Single and/or Co-Exposures on PRC2 and H3K27me3 Protein Expression Levels

**Figure 7:**
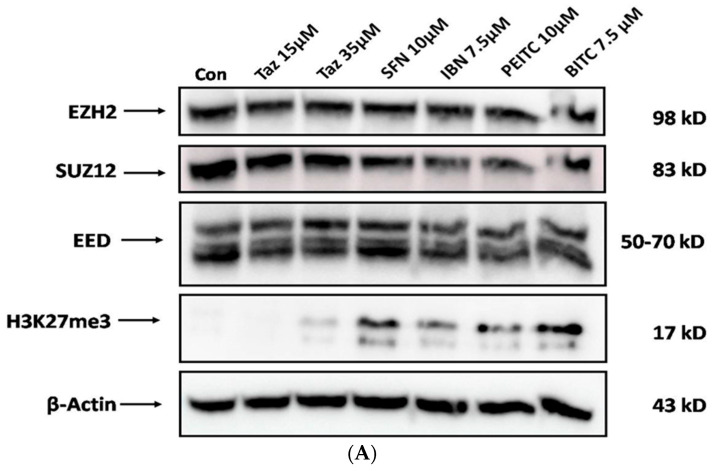

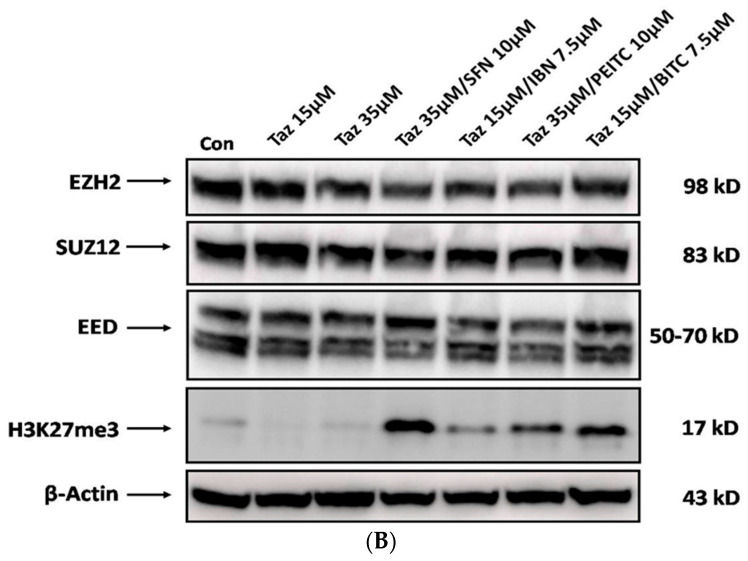
The effect of TAZ and ITCs alone or in combination on protein expression of PRC2 members and H3K27me3 content levels in malignant melanoma cells. A375 cells were subjected to either TAZ or each ITC alone (**A**) or in combination (**B**) for 48 h. Western immunoblotting analysis for EZH2, SUZ12, EED, and H3K27me3 protein expression levels. Western blots shown are representatives from two independent experiments. Stripping and re-probing the same membranes with β-actin verified equal protein loading.

Finally, we evaluated the effect of TAZ as either a single agent or in co-exposures with each ITC on EZH2, EED, and SUZ12, as well as H3K27me3 protein expression levels. Western blot analyses revealed no significant changes in the expression levels of EZH2 and EED but a much more significant reduction in the case of SUZ12 when exposed to either TAZ or each ITC alone. In addition, TAZ was capable of reducing H3K27me3 expression levels, while surprisingly exposures to each class of ITCs resulted in increased protein expression levels of H3K27me3 (Figure 7A). However, in the case of co-exposures, the levels of EED and SUZ12 remained unchanged, but those of EZH2 appeared to be reduced. Finally, a further increase in H3K27me3 expression levels was noted in the case of co-exposures with SFN and BITC, when compared to co-exposures of TAZ with IBN and PEITC (Figure 7B).

## 3. Discussion

The importance of epigenetic alterations not only allows us to identify the underlying mechanisms involved in melanoma pathophysiology but also enables us to develop more efficient therapeutic strategies based on their reversible nature via the use of epi-drugs [5,18]. Although in recent years, an increased number of epi-drugs has been developed, only a limited number has been introduced to the clinical practice, providing a significant therapeutic potential mainly against hematological malignancies [19,20]. As such, there has been an increasing interest in the use of phytochemicals as novel therapeutic agents [21,22]. In this context, several studies have demonstrated a variety of natural bioactive compounds capable of targeting the epigenome thereby altering gene expression and thus providing a therapeutic benefit in cancer treatment. To this end, combinational treatments of synthetic epigenetic inhibitors with bioactive phytochemicals appear as a promising “alternative approach” in cancer management and treatment, as opposed to monotherapy [18,23]. Among different phytochemicals, various types of ITCs have been shown to exhibit considerable activity against various epigenetic processes by restoring a normal epigenetic landscape and ultimately preventing tumor progression, including melanoma [12,24]. Based on the above characteristics, our initial aim was to evaluate the anti-cancer effect of TAZ as a single agent, as well as in co-exposures with various ITCs, against an in vitro model of human malignant melanoma. Although the cytotoxic action of TAZ has been previously studied in several types of solid tumors [25,26], limited studies have indicated its cytotoxic effect against melanoma [9]. On the other hand, the anti-melanoma effect of TAZ, as a single agent, was potentiated in co-exposure protocols with various ITCs (e.g., BITC, SFN, PEITC, and IBN) where the type of interaction varied according to the type of ITC used. Our data revealed that co-exposures between TAZ and SFN or PEITC induced a synergistic effect, whereas co-exposures between TAZ and IBN or BITC induced an additive interaction. Moreover, our data revealed TAZ-induced apoptosis to be at least 2-fold potentiated during co-exposures with each ITC, together with significant alterations in the expression of genes involved in the intrinsic and/or extrinsic apoptotic pathways. To this end, the role of both TAZ and ITCs in apoptotic induction has been previously reported in various studies utilizing different cancer models. For instance, TAZ-induced inhibition of EZH2 was shown to cause apoptotic activation in non-Hodgkin lymphoma cells [27]. On the other hand, the dual inhibition of EZH2 and Bcl-2 (by TAZ and Venetoclax, respectively) exerted a synergistic effect in mediating the apoptotic response in diffuse large B-cell lymphoma, while in the same context, TAZ was shown to induce cell cycle arrest and apoptosis against lymphoma cells in preclinical models [28,29]. In addition, ITC-induced apoptosis has been linked with altered expression patterns of various pro- and anti-apoptotic molecules. For example, exposure to PEITC inhibited the expression levels of Bcl-2 and Bcl-XL, while Bak was increased in human pancreatic cancer cells. Moreover, exposures to SFN and PEITC induced protein expression levels of p53, Bax, and caspase-3, leading to apoptotic induction in Jurkat T-leukemia cells, while apoptosis activation via increased ROS production was observed in cervical cancer cells [30,31,32]. Finally, SFN-mediated apoptosis involved upregulation of Bax and decreased expression of Bim in non-small cell lung cancer cells [33], while the activation of caspases-8, 9, 4, 3, 7, 6 was observed in melanoma cells exposed to SFN, PEITC, and BITC [34].

One important target in the epigenetic therapy of melanoma is EZH2. For example, EZH2 overexpression was shown to repress E-cadherin expression, ultimately leading to Epithelial-Mesenchymal Transition (EMT), which, together with the repression of AMD1, negatively regulates EMT and consequently metastasis [10]. Moreover, in an in vitro model of mouse cutaneous melanoma, pharmacological inhibition of EZH2 significantly reduced melanoma progression and decreased invasiveness, thereby leading to increased survival [35]. Our results revealed that exposure to TAZ or each of the ITCs alone slightly reduced the protein expression levels of SUZ12 only, while those of H3K27me3 were significantly reduced when exposed to only TAZ. However, co-exposure of TAZ with each ITC did not significantly alter the protein expression levels of any of the PRC2 family members, although H3K27me3 levels were further increased when co-exposed to TAZ with all tested ITCs. Although SFN, PEITC, and BITC were previously shown to target the aberrant epigenetic landscape of several types of cancer, including melanoma, the literature relevant to IBN’s involvement is rather limited [12]. For instance, in a recent study, IBN (as well as SFN) were shown to possess anti-melanoma activity via the reduction in histone deacetylase activity, modulation of various HDACs, methyl-, and acetyl-transferases, thereby altering the methylation and acetylation status of distinct lysine residues on H3 and H4 histones [16]. Given that gene silencing is a rather complex process that involves not only the deposition of H3K27me3 but also the recruitment of various other key epigenetic players (such as HDACs and DNMTs), it becomes apparent that any potential role of ITCs in inducing the re-expression of previously repressed apoptotic genes needs to be further investigated. Finally, it is also intriguing as to how significantly increased levels of H3K27me3 levels occur in the presence of changes in the protein expression levels of the PRC2 family members and especially that of EZH2, at conditions where TAZ is co-exposed to ITCs, particularly SFN or BITC. To these ends, a better understanding of how SFN and BITC induce TAZ’s potentiation of apoptosis is of paramount importance. 

## 4. Materials and Methods

### 4.1. Cell Lines and Culture Conditions

A375 cells (non-metastatic melanoma) were purchased from the American Type Culture Collection (ATCC—Manassas, VA, USA), while Colo-679 (metastatic melanoma) cells were purchased from DeutchSammlung von Mikroorganismen und Zellkultuten (DSMZ—Braunscgweig, Germany). HaCaT (immortalized non-tumorigenic keratinocytes) cells were kindly provided by Dr. Sharon Broby (Dermal Toxicology & Effects Group; Centre for Radiation, Chemical and Environmental Hazards; Public Health England, London, UK). All cell lines were tested for mycoplasma contamination and authenticated using the STR method. A375 and HaCaT cells were maintained in DMEM high glucose media, while Colo-679 was maintained in RPMI-1640 media (Biosera, Kansas City, MO, USA). Cells were supplemented with 10% FBS, 1% pen/strep (100 U/mL penicillin and 100 μg/mL streptomycin), and 2 mM L-glutamine (Biosera, Kansas City, MO, USA) and maintained in a humidified incubator at 37 °C and 5% CO_2_. Cells were grown as monolayers and subcultured when they reached 80–90% confluence. A375 and Colo-679 were utilized for the purpose of demonstrating the therapeutic efficacy of TAZ, while HaCaT were utilized as melanoma-neighboring keratinocyte cells to demonstrate the lack of TAZ-induced side toxicity (often associated with many treatment protocols) in our experiments. In all experimental procedures, 0.1% of DMSO was utilized (as the organic solvent used for diluting TAZ and each of the ITCs) in order to demonstrate a lack of toxicity due to non-specific DMSO-induced toxicity.

### 4.2. Determination of Cell Viability

At the end of all exposure conditions, cell viability was determined by the Alamar Blue assay and expressed as a percentage of untreated (control) cells, while 0.1% DMSO was used as a negative control in our experiments.

### 4.3. Exposure Protocols

A375 cells were subjected to increasing concentrations (30–40 μM) of TAZ as a single agent (24–72 h of exposure) while respective EC_50_ values were calculated using the CompuSyn software, V.20 (Biosoft, Cambridge, UK). 

In another set of experiments, A375 cells were exposed to various combinational conditions of TAZ with each of the ITCs over 48 h of exposure. Specifically, a range of different EC_50_ values of TAZ (Selleckchem, Houston, TX, USA) were combined with pre-determined EC_50_ values of SFN, IBN (Abcam, Cambridge, UK), PEITC and BITC (Sigma-Aldrich, St. Louis, MO, USA) as previously described [15,16]. Consequently, combinatorial treatments of TAZ with each of the ITCs were evaluated as follows: (i) 100% of the EC_50_ of TAZ with 100% of the EC_50_ of each ITC; (ii) 75% of the EC_50_ of TAZ with 75% of the EC_50_ of each ITC; (iii) 50% of the EC_50_ of TAZ with 50% of the EC_50_ of each ITC; and (iv) 25% of the EC_50_ of TAZ with 25% of the EC_50_ of each ITC (Exposure Protocol 1) (Figure S1).

Next, new EC_50_ values for both TAZ and different ITCs were selected by means of maintaining a 50% reduction in viability levels of A375 cells. Consequently, A375 cells were subjected to combined exposures consisting of (i) gradually decreasing concentrations of the selected EC_50_ of TAZ together with (ii) gradually increasing concentrations of the EC_50_ of each ITC (Exposure Protocol 2) (Figure S2). Combined concentrations of TAZ with each ITC (capable of causing a reduction in cell viability levels between 45 and 65%) were also used for exposing HaCaT cells (Table S1). Finally, combinational conditions (that caused a 50% decrease in cell viability in A375 and Colo-679 while maintaining it at over 80% in HaCaT cells) were selected as optimized exposure conditions in all subsequent experiments described thereafter. Specifically, our optimized combinational protocol consisted of the following experimental conditions: (i) 35 μM of TAZ + 10 μM SFN; (ii) 15 μM TAZ + 7.5 μM IBN; (iii) 35 μM TAZ + 10 μM PEITC; and (iv) 15 μM TAZ + 7.5 μM BITC.

### 4.4. Combination Index Analyses

Combination index analyses were performed using the CompuSyn software, V.20 (Biosoft, Cambridge, UK) (Figure S3). According to the Chou–Talalay method, the interactions between TAZ and each ITC were identified by calculating the Combination Index (CI), which is considered the standard measure of combinatorial effect(s) [36,37,38].

### 4.5. Determination of Caspase-3 Activity Levels

Measurements were performed by utilizing the fluorometric caspase-3, -8, and -9 Multiplex Activity Assay Kit (Abcam, Cambridge, UK) as per the manufacturer’s instructions. The activity of caspase-3 was monitored in a fluorescence microplate reader (Synergy H1, Bio-Tek, Agilent, Santa Clara, CA, USA) at 535/620 nm. 

### 4.6. Real-Time Polymerase Chain Reaction (RT-PCR) Protocol

Extraction of RNA was performed with the use of a NucleoZOL reagent (Macherey-Nagel, Duren, Germany). RNA quality was determined with NanoDrop One/OneC (Thermo Scientific, Waltham, MA, USA), whereas PrimeScript™ First Strand cDNA Synthesis kit (TaKaRa, Dalian, China) was used for the synthesis of the complementary DNA (cDNA). RT-PCR experiments were performed with the StepOne Plus RT-PCR system (Applied Biosystems, Carlsbad, CA, USA) using SYBR Green qPCR mastermix (MedChem Tronica, Sollentura, Sweeden). The sequences of forward and reverse primers were purchased from Invitrogen, Thermo-Fischer Scientific (Waltham, MA, USA), and are listed in Table S4. Finally, gene expression levels were normalized to *β-actin* and expressed as fold-change compared to control (untreated) samples, using the 2^−ΔΔCt^ method. 

### 4.7. Western Immunoblotting Protocol

PBS-washed cells were lysed and then incubated for 30 min at 4 °C for preparation of whole cell extracts. Determination of protein concentration was performed by utilizing the Pierce™ BCA Protein Assay kit (Thermo Scientific, Rockford, IL, USA). Whole-cell extracts were subjected to SDS-PAGE and then transferred on PVDF membranes (Millipore, Bedford, MA, USA), which were incubated overnight at 4 °C with primary antibodies against anti-EZH2 (1/1000), anti-EED (1/1000), anti-SUZ12 (1/2000), anti-tri-methyl Histone H3 (Lys 27) (1/1000) (Cell Signaling Technology, Boston, MA, USA) and anti-β-actin (1/20,000) (Sigma-Aldrich, Taufkirchen, Germany). The next day, membranes were incubated with the appropriate secondary antibody (Millipore, Bedford, MA, USA). Protein bands were developed using a chemiluminescent substrate (Thermo Scientific, Rockford, IL, USA), and images were obtained with a Vilber FUSION Solo 6X imager (Vilber Lourmat, Collégien, France). Blots were stripped and re-probed with an appropriate antibody. Stripping and re-probing of membranes with anti-β-actin antibody was used to ensure equal protein loading. 

### 4.8. Statistical Analyses 

Data were expressed as mean values ± standard error of the mean (SEM), and comparisons were performed between untreated (control) and treated samples. Statistical analyses were performed by one-way ANOVA with Tukey’s test for multiple comparisons, with the use of Graph Pad Prism 5.0 (GraphPad Software, San Diego, CA, USA). Values of *p* < 0.05, *p* < 0.01, and *p* < 0.001 were considered statistically significant.

## 5. Conclusions

We developed an experimental protocol based on novel combinational conditions, providing evidence of its therapeutic efficacy. Specifically, via the use of different types of ITCs, we were able to enhance the anti-melanoma activity of the clinical epigenetic drug TAZ by maintaining its specificity and therapeutic efficacy against human malignant melanoma cells while at the same time maintaining minimal toxicity in neighboring non-tumorigenic keratinocyte cells. Furthermore, we documented the effect of our combined experimental protocols in altering the expression levels of some of the PRC2 complex members, as well as the expression levels of H3K27me3. 

## Figures and Tables

**Table 1:** Interaction analysis between TAZ and each of the ITCs in A375 cells. Analysis was performed by the Chou–Talalay technique, and all potential interactions were determined by calculating the Combination Index. The CompuSyn software, V.20 (Biosoft, Cambridge, UK), was utilized where CI < 1 indicates a synergistic, CI = 1 an additive, and CI > 1 an antagonistic interaction.

Combinatorial Exposure	Combination Index (CI)	Agent Interaction
TAZ (35.0 μM) + SFN (10.0 μM)	0.89	Synergistic
TAZ (15.0 μM) + IBN (7.5 μM)	1.02	Additive
TAZ (35.0 μM) + PEITC (10.0 μM)	0.79	Synergistic
TAZ (15.0 μM) + BITC (7.5 μM)	1.09	Additive

## Data Availability

The datasets used and/or analyzed during the current study are available from the corresponding author upon reasonable request.

## Supplementary Materials

The following supporting information can be downloaded at https://www.mdpi.com/article/10.3390/ijms25052745/s1.
