# Supplementary material for: Isothiocyanates Potentiate Tazemetostat-Induced Apoptosis by Modulating the Expression of Apoptotic Genes, Members of Polycomb Repressive Complex 2, and Levels of Tri-Methylating Lysine 27 at Histone 3 in Human Malignant Melanoma Cells
Source: Int J Mol Sci. 2024 Feb 27;25(5):2745. doi: 10.3390/ijms25052745 (PMC10931595; doi:10.3390/ijms25052745)
Supplement: Supplementary file 1 [file ijms-25-02745-s001.zip › ijms-2860373-supplementary.pdf]

Supplementary Data

Isothiocyanates potentiate Tazemetostat-induced apoptosis by modulating the expression of apoptotic genes, members of Polycomb Repressive Complex 2 and levels of tri-methylating lysine 27, at Histone 3, in human malignant melanoma cells

Ioannis Anestopoulos[1†], Ioannis Paraskevaidis[1†], Sotiris Kyriakou[1], Lambrini Giova[1], Dimitrios T. Trafalis[2], Sotiris Botaitis[3], Rodrigo Franco[4,5], Aglaia Pappa[6], Mihalis I. Panayiotidis[1#]

[1]Department of Cancer Genetics, Therapeutics & Ultrastructural Pathology, The Cyprus Institute of Neurology & Genetics, Nicosia 2371, Cyprus; [2]Laboratory of Pharmacology, Medical School, National & Kapodistrian University of Athens, Athens 11527, Greece; [3]Department of Surgery, University Hospital, Democritus University of Thrace, School of Medicine, Alexandroupolis68100, Greece; [4]School of Veterinary Medicine & Biomedical Sciences and [5]Redox Biology Centre, University of Nebraska-Lincoln, Nebraska 68583, USA; [6]Department of Molecular Biology & Genetics, Democritus University of Thrace, Alexandroupolis 68100, Greece

[†]These authors contributed equally to this work

[#]**Corresponding author:** Prof. Dr. Mihalis I. Panayiotidis, Department of Cancer Genetics, Therapeutics &Ultrastructural Pathology, The Cyprus Institute of Neurology & Genetics, 6 Iroon Avenue, Ayios Dometios, Nicosia 2371, Cyprus. E-mail: mihalisp@cing.ac.cy

Figure S1

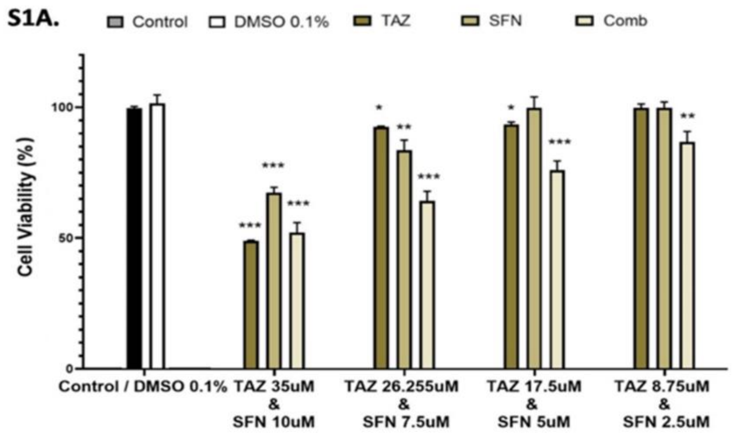
S1A.
Control
DMSO 0.1%
TAZ
SFN
Comb
Cell Viability (%)
100
50
0
***
***
***
*
**
***
*
***
**
Control / DMSO 0.1%
TAZ 35uM & SFN 10uM
TAZ 26.255uM & SFN 7.5uM
TAZ 17.5uM & SFN 5uM
TAZ 8.75uM & SFN 2.5uM

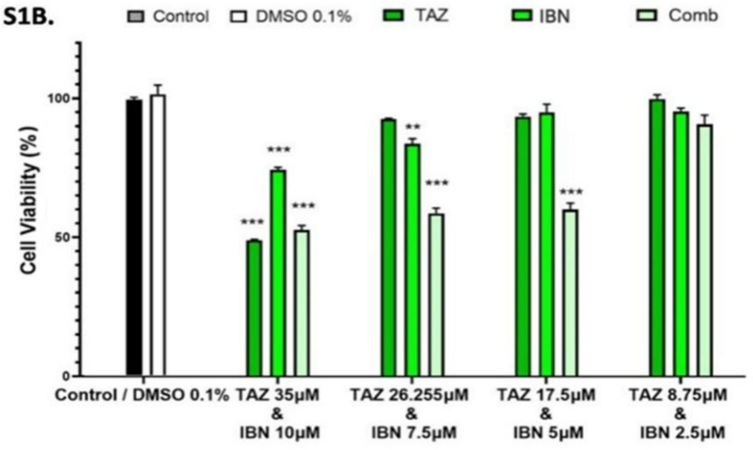
S1B.
Control
DMSO 0.1%
TAZ
IBN
Comb
Cell Viability (%)
100
50
0
***
***
***
**
***
***
Control / DMSO 0.1%
TAZ 35μM & IBN 10μM
TAZ 26.255μM & IBN 7.5μM
TAZ 17.5μM & IBN 5μM
TAZ 8.75μM & IBN 2.5μM

S1C.

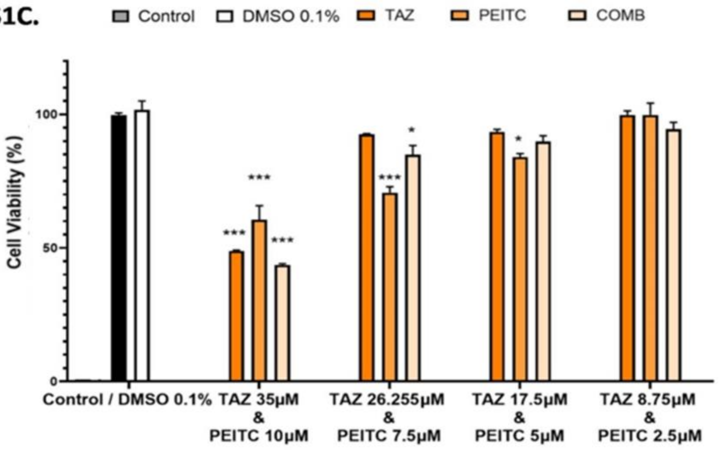

S1D.

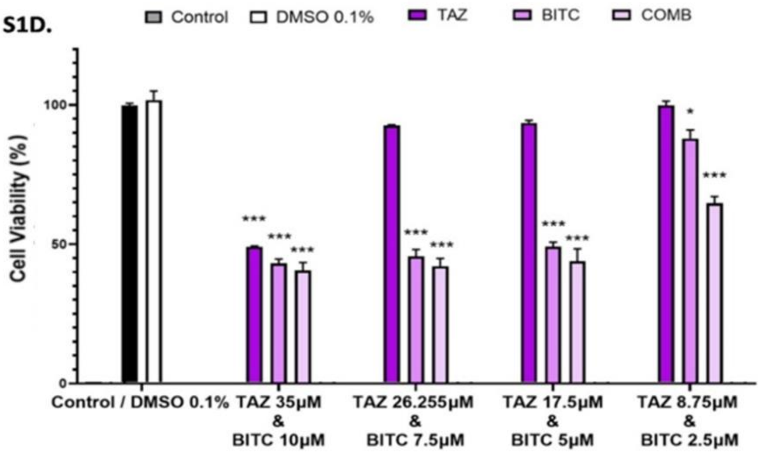

Cell viability levels of A375 cells under Exposure Protocol 1. Cell viability levels of A375 cells were recorded following different exposure conditions between TAZ alone, each ITC alone or between TAZ combined with SFN (S1A), IBN (S1B), PEITC (S1C) and BEITC (S1D). Specifically, combinational exposure protocols between TAZ and each ITC were applied as follows: i) 100% the EC_{50} concentration of TAZ (35μM) : 100% the EC_{50} value of each ITC (10μM); ii) 75% of the EC_{50} concentration of TAZ (26.255μM) : 75% of EC_{50} value of each ITC (7.55μM); iii) 50% of EC_{50}concentration of TAZ (17.5μM) : 50% of EC_{50} value of each ITC (5μM); iv) 25% of EC_{50} concentration of TAZ (6.75μM) : 25% of EC_{50} value of each ITC (2.5μM). Cell viability was determined by using the Alamar Blue assay. All data are expressed as means of five replicates ± SEM and are representative of three independent experiments. Statistical significance is indicated by *$p<0.05$, **$p<0.01$, ***$p<0.001$ relative to the corresponding 0.1% DMSO controls.

Figure S2

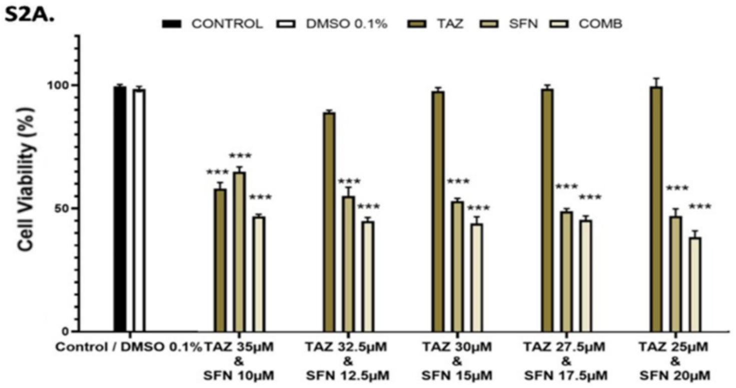
S2A.
CONTROL
DMSO 0.1%
TAZ
SFN
COMB
Cell Viability (%)
100
50
0
***
Control / DMSO 0.1%
TAZ 35µM & SFN 10µM
TAZ 32.5µM & SFN 12.5µM
TAZ 30µM & SFN 15µM
TAZ 27.5µM & SFN 17.5µM
TAZ 25µM & SFN 20µM

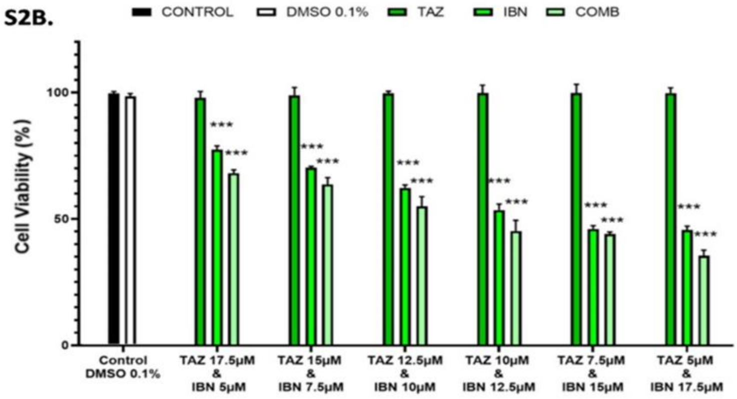
S2B.
CONTROL
DMSO 0.1%
TAZ
IBN
COMB
Cell Viability (%)
100
50
0
***
Control DMSO 0.1%
TAZ 17.5µM & IBN 5µM
TAZ 15µM & IBN 7.5µM
TAZ 12.5µM & IBN 10µM
TAZ 10µM & IBN 12.5µM
TAZ 7.5µM & IBN 15µM
TAZ 5µM & IBN 17.5µM

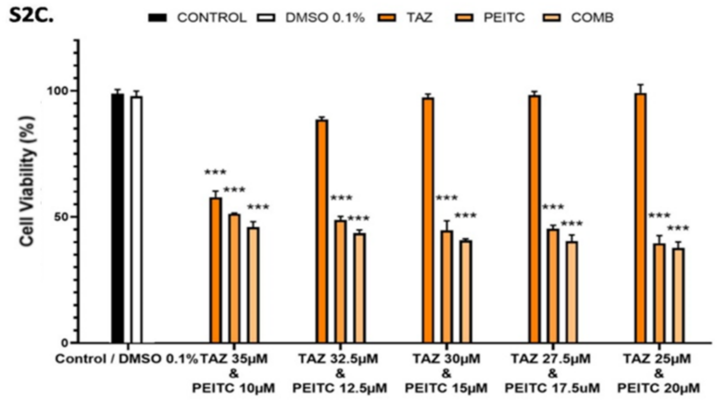

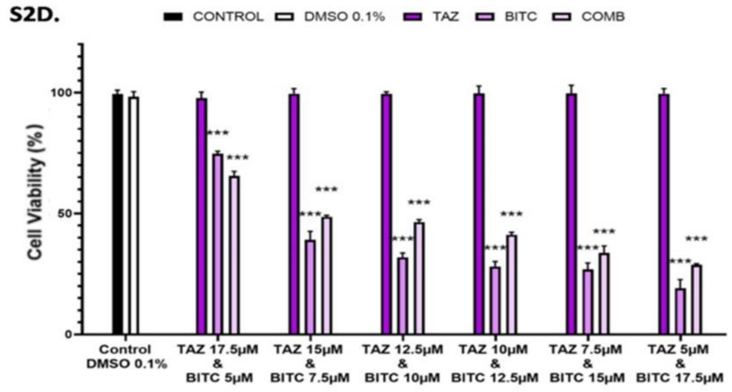

Cell viability levels of A375 cells under Exposure Protocol 2. Cell viability levels of A375 cells were recorded following different exposure conditions between TAZ alone, each ITC alone or between TAZ combined with SFN (S2A), IBN (S2B), PEITC (S2C) and BEITC (S2D). Specifically, A375 cells were exposed to gradually decreasing concentrations of the EC_{50} concentration of TAZ (by 2.5μM) together with a parallel gradual increase of the EC_{50} concentrations of each ITC (by 2.5μM) until reached a final concentration of 20μM, at the most. All data are expressed as means of five replicates ± SEM and are representative of three independent experiments. Statistical significance is indicated by *p<0.05, **p<0.01, ***p<0.001 relative to the corresponding 0.1% DMSO controls.

Figure S3

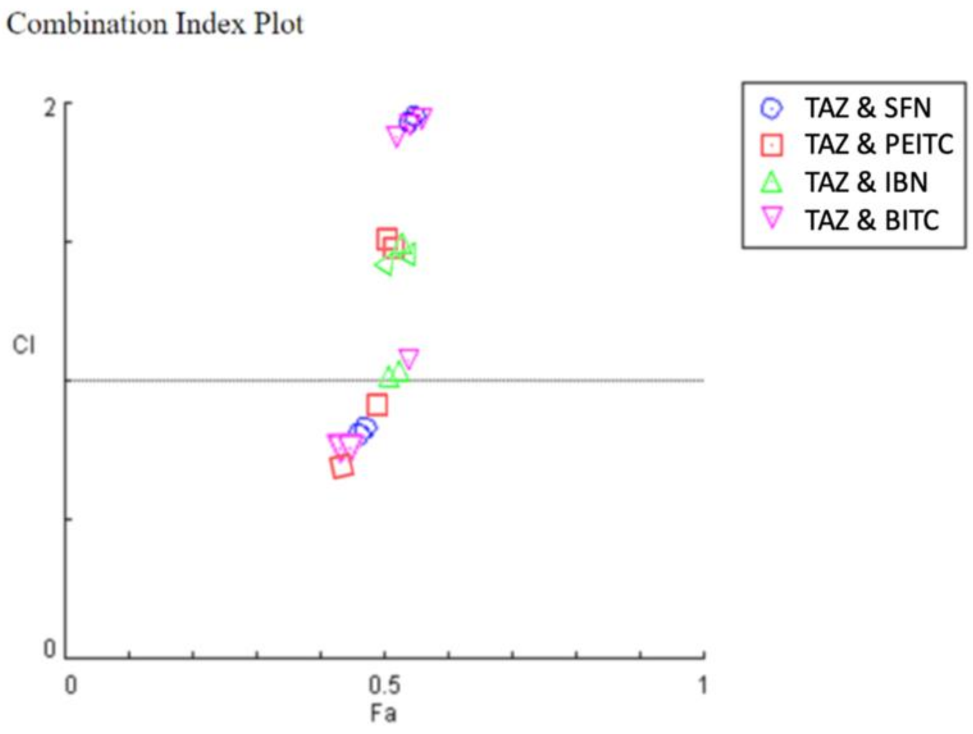

Combination Index plot of combinatorial treatments of TAZ with different ITCs in A375 cells. Combination Index (CI) plot descriptive of the combinatorial exposure of TAZ with each ITC against A375 melanoma cells. The interaction of each individual combinatorial exposure was measured using the CompuSyn software, V.20 (Biosoft, Cambridge, UK) where CI<1 is descriptive of a synergistic, CI=1 an additive and CI>1 an antagonistic interaction.

Figure S4.

S4A. Isobologram TAZ+SFN

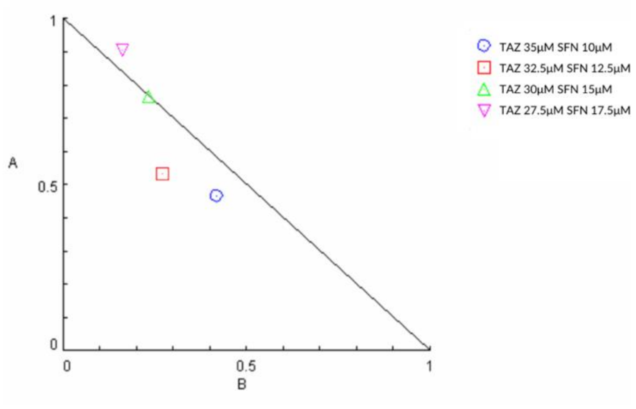

S4B. Isobologram TAZ+IBN

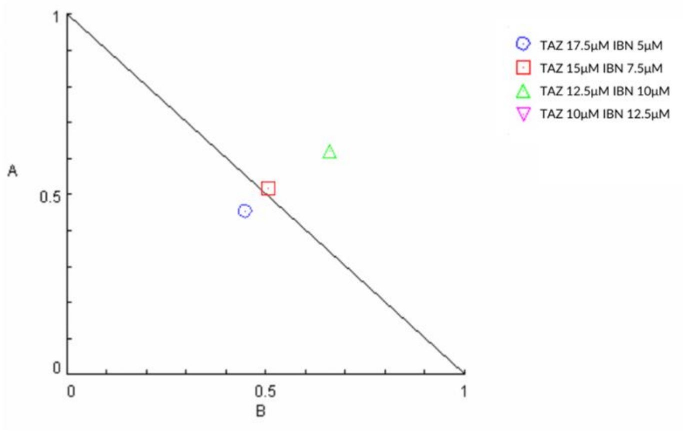

S4C. Isobologram TAZ+PEITC

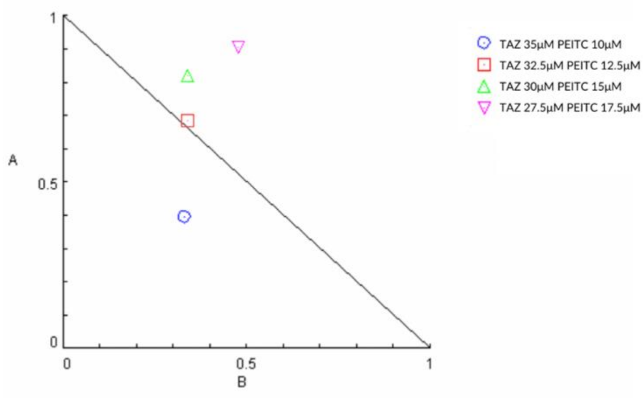

S4D. Isobologram TAZ+BITC

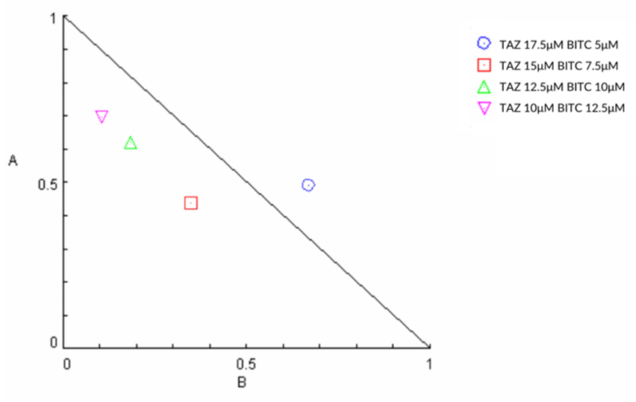

Normalized isobolograms showing the interaction between TAZ and different ITCs in inhibiting cell growth of A375 melanoma cells. Isobologram analysis was based on the results of cell viability, following treatments of A375 cells with combined exposure conditions of TAZ and SFN (A), IBN (B), PEITC (C) and BITC (D), according to experimental protocol 2 (**Fig. S2**). Combination data points that are below the line are synergistic, above the line are antagonistic and on the line are additive. The interaction of each individual combinatorial exposure was measured using the CompuSyn software, V.20 (Biosoft, Cambridge, UK).

Table S1. Cell viability levels following various combinatorial exposure protocols with TAZ and each of the ITCs in HaCaT cells. The rationale for the indicated combinatorial exposure conditions was based on their efficacy to reduce the viability levels of A375 cells around their EC_{50} value (45-65%) while inducing a safe cytotoxic profile against HaCaT cells (over 80%). To this end, bold highlighted conditions were selected, as optimum, and utilized in the next series of experiments. All data are expressed as means of five replicates ± SEM and are representative of three independent experiments.

**EXPOSURE PROTOCOL**	**CELL VIABILITY LEVELS**
Control	100.00 ± 2.89
0.1% DMSO	98.10 ± 2.32
**TAZ (35.0μM)+ SFN (10.0μM)**	**86.51 ± 2.00**
TAZ (32.5μM)+ SFN (12.5μM)	79.37 ± 0.90
TAZ (30.0μM)+ SFN (15.0μM)	63.01 ± 3.19
**TAZ (15.0μM)+ IBN (7.5μM)**	**90.12 ± 1.85**
TAZ (12.5μM)+ IBN (10.0μM)	79.39 ± 1.63
TAZ (10.0μM)+ IBN (12.5μM)	72.34 ± 0.97
**TAZ (35.0μM)+ PEITC (10.0μM)**	**91.11 ± 1.80**
TAZ (32.5μM)+ PEITC (12.5μM)	75.01 ± 3.23
TAZ (30.0μM)+ PEITC (15.0μM)	69.06 ± 4.28
**TAZ (15.0μM)+ BITC (7.5μM)**	**82.45 ± 2.97**

TAZ (12.5μM)+ BITC (10.0μM)	79.55 ± 4.00
TAZ (10.0μM)+ BITC (12.5μM)	73.62 ± 4.06

Table S2. Fold change in expression levels of genes involved in intrinsic and extrinsic apoptosis in A375 cells exposed, for 48h, to TAZ as a single agent and/or in combined exposures with each ITC. Fold changes in gene expression levels were determined by Real-Time PCR (RT-PCR). All data are expressed as means of three replicates ± SEM and are representative of three independent experiments. Gene expression data were normalized to β-actin, using the 2-ΔΔCt method and were expressed as fold-change compared to untreated (control) samples.

**INTRINSIC PATHWAY**					
	**TAZ**	**TAZ+SFN**	**TAZ+IBN**	**TAZ+PEITC**	**TAZ+BITC**
CASP3	**1.515**±0.019	**1.888**±0.052	**1.78**±0.001	**1.852**±0.002	**1.81**±0.068
BID	**0.99**±0.020	**1.114**±0.033	**1.337**±0.038	**1.08**±0.032	**1.576**±0.002
BAX	**2.44**±0.006	**2.062**±0.080	**1.752**±0.067	**1.83**±0.012	**2.16**±0.002
XIAP	**0.89**±0.001	**0.59**±0.029	**0.742**±0.137	**0.84**±0.210	**0.705**±0.190
PMAIP1	**1.57**±0.900	**1.0877**±0.012	**1.147**±0.058	**0.942**±0.002	**1.212**±0.015
BAD	**1.24**±1.160	**2.539**±0.305	**4.635**±0.002	**2.1**±0.342	**1.786**±0.642
CYCS	**1.22**±0.009	**1.314**±0.122	**1.348**±0.002	**1.42**±0.124	**1.568**±0.095
BAK1	**5.064**±0.021	**2.26**±0.860	**5.301**±0.127	**1.19**±0.089	**3.17**±0.011
APAF1	**4.992**±0.032	**5.902**±0.100	**5.121**±0.100	**6.21**±0.901	**6.121**±0.567
CASP9	**1.229**±0.403	**1.166**±0.089	**1.786**±0.040	**1.09**±0.003	**1.642**±0.012
CASP2	**2.745**±0.395	**2.37**±0.036	**2.194**±0.025	**2.42**±0.213	**2.173**±0.075
DIABLO (SMAC)	**1.896**±0.058	**1.075**±0.019	**1.608**±0.007	**1.103**±0.013	**1.426**±0.008
BCL2L11 (BIM)	**0.082**±0.009	**1.371**±0.076	**1.355**±0.124	**1.124**±0.035	**1.310**±0.039
CASP6	**1.494**±0.038	**2.063**±0.256	**2.495**±0.155	**2.032**±0.421	**5.211**±0.081
CASP7	**1.05**±0.102	**1.103**±0.562	**1.058**±0.139	**1.432**±0.242	**1.037**±0.024
MCL1	**0.104**±0.021	**0.592**±0.066	**0.077**±0.039	**0.720**±0.042	**0.966**±0.064

BCL2	**1.029**±0.030	**1.016**±0.480	**0.980**±0.020	**1.092**±0.132	**1.033**±0.052
BCL2L1	**0.439**±0.039	**0.972**±0.003	**1.099**±0.031	**0.801**±0.002	**0.745**±0.0355

**EXTRINSIC PATHWAY**					
	**TAZ**	**TAZ+SFN**	**TAZ+IBN**	**TAZ+PEITC**	**TAZ+BITC**
FASL	**1.102**±0.096	**1.002**±0.515	**1.003**±0.095	**1.142**±0.002	**1.022**±0.351
FAS	**1.375**±0.124	**1.039**±0.742	**1.393**±0.145	**1.462**±0.075	**0.879**±0.052
FAIM	**1.045**±0.040	**0.838**±0.481	**0.916**±0.145	**0.920**±0.003	**0.913**±0.012
TNFRSF10 (TRAIL)	**2.107**±0.415	**1.612**±0.762	**6.268**±0.081	**1.529**±0.052	**1.990**±0.137
TNFRSF10D	**0.745**±0.395	**0.837**±0.036	**0.794**±0.025	**0.842**±0.213	**0.873**±0.075
TNFRSF10C	**0.839**±0.162	**0.836**±0.014	**0.907**±0.163	**0.739**±0.034	**0.943**±0.005
TNFRSF10CB	**1.048**±0.325	**1.037**±0.021	**1.065**±0.133	**1.092**±0.031	**0.913**±0.061
TNFRSF10A	**1.622**±0.001	**1.348**±0.047	**1.160**±0.070	**1.423**±0.004	**1.450**±0.091
FADD	**1.102**±0.001	**1.068**±0.133	**1.047**±0.120	**1.342**±0.103	**1.052**±0.188
CASPASE8	**1.57**±0.900	**1.877**±0.012	**1.547**±0.058	**1.942**±0.002	**1.612**±0.015
CASPASE 10	**1.22**±0.009	**1.314**±0.122	**1.348**±0.002	**1.420**±0.124	**1.568**±0.095
C-FLAR	**0.351**±0.075	**0.303**±0.009	**0.406**±0.049	**0.323**±0.122	**0.461**±0.026
TNF	**1.657**±0.463	**2.147**±0.038	**2.090**±0.020	**2.682**±0.001	**1.583**±0.354
TNFRSF1A (TNFR1)	**1.55**±0.928	**9.143**±0.051	**5.214**±0.008	**5.130**±0.053	**4.655**±0.020
TRADD	**4.992**±0.032	**5.902**±0.100	**5.121**±0.100	**6.210**±0.901	**6.121**±0.567
TRAF2	**3.252**±0.347	**1.331**±0.054	**2.514**±0.028	**1.582**±0.042	**1.092**±0.055
BIRC2 (CIAP1)	**1.412**±0.026	**0.795**±0.074	**0.825**±0.024	**0.069**±0.120	**0.610**±0.077
BIRC3 (CIAP2)	**0.676**±0.054	**0.495**±0.174	**0.401**±0.174	**0.492**±0.005	**0.592**±0.303
TRAF1	**0.821**±0.141	**0.992**±0.050	**1.010**±0.625	**1.021**±0.013	**1.272**±0.426
TRAF5	**1.44**±0.699	**5.469**±0.403	**3.855**±0.658	**5.992**±0.341	**1.405**±0.017
TNFAIP3/A20	**0.902**±0.001	**0.899**±0.029	**0.842**±0.137	**0.732**±0.210	**0.705**±0.190

Table S3. Fold change in expression levels of genes involved in intrinsic and extrinsic apoptosis in Colo-679 cells exposed, for 48h, to TAZ as a single agent and/or in combined exposures with each ITC. Fold changes in gene expression levels were determined by Real-Time PCR (RT-PCR). All data are expressed as means of three replicates ± SEM and are representative of three independent experiments. Gene expression data were normalized to β-actin, using the 2-ΔΔCt method and were expressed as fold-change compared to untreated (control) samples.

**INTRINSIC PATHWAY**					
	**TAZ**	**TAZ+SFN**	**TAZ+IBN**	**TAZ+PEITC**	**TAZ+BITC**
CASP3	**1.219**±.0.031	**1.530**±0.014	**1.359**±0.032	**1.629**±0.328	**1.721**±0.032
BID	**1.241**±0.039	**1.429**±0.039	**1.429**±0.012	**1.420**±0.932	**1.672**±0.084
BAX	**1.924**±0.042	**1.948**±0.029	**1.602**±0.892	**2.019**±0.382	**1.982**±0.042
XIAP	**1.121**±0.029	**0.913**±0.079	**0.929**±0.249	**0.719**±0.029	**0.902**±0.249
PMAIP1	**1.103**±0.204	**1.231**±0.029	**1.001**±0.039	**1.429**±0.329	**1.182**±0.092
BAD	**0.924**±0.021	**2.012**±0.039	**2.952**±0.042	**2.443**±0.042	**1.292**±0.034
CYCS	**1.029**±0.029	**1.210**±0.082	**1.492**±0.095	**1.329**±0.320	**1.029**±0.291
BAK1	**2.293**±0.129	**2.420**±0.193	**1.234**±0.391	**1.829**±0.093	**2.219**±0.032
APAF1	**3.211**±0.013	**3.992**±0.031	**4.829**±0.009	**4.90**±0.003	**5.230**±0.002
CASP9	**1.682**±0.311	**1.428**±0.021	**1.929**±0.032	**1.429**±0.203	**1.824**±0.249
CASP2	**1.839**±0.049	**3.572**±0.103	**2.453**±0.042	**2.824**±0.192	**3.529**±0.127
DIABLO (SMAC)	**1.244**±0.049	**1.294**±0.294	**1.432**±0.029	1.320±0.032	**1.120**±0.039
BCL2L11 (BIM)	**0.903**±0.024	**1.423**±0.049	**1.329**±0.082	**1.439**±0.049	**1.728**±0.039
CASP6	**1.210**±0.042	**1.932**±0.194	**1.329**±0.032	**1.235**±0.241	**2.103**±0.391
CASP7	**1.330**±0.432	**1.492**±0.239	**1.029**±0.029	**1.782**±0.092	**1.324**±0.042

MCL1	**0.429**±0.249	**0.892**±0.023	**0.530**±0.040	**0.920**±0.093	**1.103**±0.078
BCL2	**1.392**±0.204	**1.284**±0.029	**1.392**±0.920	**1.923**±0.033	**1.299**±0.029
BCL2L1	**0.892**±0.042	**0.823**±0.320	**0.929**±0.194	**0.702**±0.320	**1.203**±0.013

**EXTRINSIC PATHWAY**					
	**TAZ**	**TAZ+SFN**	**TAZ+IBN**	**TAZ+PEITC**	**TAZ+BITC**
FASL	**1.209**±0.024	**1.382**±0.329	**1.214**±0.029	**1.492**±0.492	**1.102**±0.293
FAS	**1.637**±0.294	**1.392**±0.424	**1.628**±0.049	**1.104**±0.029	**1.210**±0.091
FAIM	**1.103**±0.043	**1.121**±0.032	**0.820**±0.140	**0.792**±0.129	**0.924**±0.029
TNFRSF10 (TRAIL)	**1.723**±0.028	**1.820**±0.293	**1.942**±0.099	**1.992**±0.320	**2.492**±0.320
TNFRSF10D	**1.102**±0.293	**1.021**±0.029	**0.920**±0.092	**0.992**±0.104	**1.201**±0.039
TNFRSF10C	**0.920**±0.439	**0.942**±0.294	**0.603**±0.240	**0.829**±0.312	**1.243**±0.021
TNFRSF10CB	**1.240**±0.421	**1.102**±0.219	**1.293**±0.320	**1.112**±0.043	**1.210**±0.091
TNFRSF10A	**1.213**±0.029	**1.429**±0.093	**1.482**±0.329	**1.392**±0.328	**1.943**±0.201
FADD	**1.204**±0.391	**1.039**±0.019	**1.102**±0.102	**1.428**±0.210	**1.129**±0.210
CASPASE8	**1.319**±0.219	**1.219**±0.013	**1.928**±0.328	**1.221**±0.039	**1.567**±0.032
CASPASE 10	**1.201**±0.032	**1.426**±0.043	**1.232**±0.094	**1.219**±0.001	**1.928**±0.210
C-FLAR	**0.582**±0.192	**0.792**±0.021	**0.672**±0.024	**0.910**±0.211	**0.674**±0.031
TNF	**1.205**±0.059	**2.882**±0.002	**2.320**±0.149	**2.253**±0.112	**1.924**±0.032
TNFRSF1A (TNFR1)	**1.832**±0.053	**3.249**±0.104	**3.243**±0.003	**4.243**±0.043	**5.252**±0.039
TRADD	**3.214**±0.104	**6.254**±0.130	**5.043**±0.129	**4.942**±0.001	**4.543**±0.018
TRAF2	**2.193**±0.021	**1.392**±0.039	**1.928**±0.049	**1.928**±0.073	**1.674**±0.023
BIRC2 (CIAP1)	**1.829**±0.037	**1.203**±0.206	**0.922**±0.042	**0.822**±0.102	**0.782**±0.001
BIRC3 (CIAP2)	**0.892**±0.002	**0.864**±0.027	**0.804**±0.094	**0.792**±0.067	**0.881**±0.012
TRAF1	**0.955**±0.032	**1.103**±0.003	**1.142**±0.032	**0.921**±0.094	**0.892**±0.074
TRAF5	**1.730**±0.004	**4.357**±0.027	**2.934**±0.003	**3.452**±0.039	**1.839**±0.092
TNFAIP3/A20	**0.993**±0.024	**0.928**±0.018	**0.911**±0.049	**0.892**±0.032	**0.983**±0.072

Table S4. List of primer sequences used in RT-PCR experiments.

GENE	FORWARD PRIMER	REVERSE PRIMER
**INTRINSIC APOPTOTIC PATHWAY**		
CASP2	5’- ATTGGATCCCTTGGGCACCTC-3’	5’ ATCATGTCTGAGCGCGTGGG-3’
CASP3	5’ TTATTCAGGCCTGCCGTGGT-3’	5’- AGCATGGCACAAAGCGACTG-3’
CASP6	5- TTTGGCTGCAATGAGCTCGG-3’	5’- GGCATCTGCGTGGCTAACAG-3’
CASP7	5’- AAGCTGAGGGAGCGTCCTAC-3’	5- ACCGGTCTGGCTTAGCATCC-3’
CASP9	5’-CGGTGACGCAAGAGCGAATC-3’	5- GATCAGCTGCCTGGCCTGAT-3’
APAF1	5’- GTTGGGTTCATGGTGTGATG-3’	5’- TTTGTCTCCCAGAGCCTGA-3’
BAD	5’- TCAGGGGCCTCGAGATCGG-3’	5’- TCCTGCTCACTCGGCTCAAA-3’
BAK1	5’- AGACCTGAAAAATGGCTTCG	5’- CGGAAAACCTCCTCTGTGTC-3’
BCL2	5’- AGTACCTGAACCGGCACCT-3’	5’- GCCGTACAGTTCCACAAAGG-3’
BID	5’- TGCAGCTCAGGAACACCA-3’	5’-TCTCCATGTCTCTAGGGTAGGC-3’
BAX	5’- ACCTTGACTTGATTAGTGCCTTCT-3’	5’- GGGTCATCAATGAACTTGAGC-3’
XIAP	5’- GCAAGAGCTCAAGGAGACCA-3’	5- AAGGGTATTAGGATGGGAGTTCA-3’
MCL1	5’- TTACGACGGGTTGGGGATGG-3’	5’- CTGCCCCAGTTTGTTACGCC-3’
CYCS	5’- TTCGGAGCGGGAGTGTTCGT-3’	5’- TGTGGCACTGGGAACACTTCAT-3’

DIABLO	5'- TGACTGCAGTTGGTCTTTCAG-3'	5'- GCGGTTATAGAGGCCTGATCT-3'
BCL2L11	5'- ACTGGAGAGCTCATTGCAGAC-3'	5'- AAATACCAGGACCCGAAGGT-3'
BCL2L1	5'- CGTGGAAAGCGTAGACAAG-3'	5'- AAGAGTGAGCCCAGCAGAA-3'
PMAIP1	5'- CAAGAACGCTCAACCGAGCC-3'	5'- AGGAGTCCCCTCATGCAAGT-3'
**EXTRINSIC APOPTOTIC PATHWAY**		
CASP8	5'- GGCGGAGGGTCGATCATCTAT-3'	5'- TTCCTTCTCCCAGGATGACCC-3'
CASP10	5'- GGTAGTAATGAGGGCAGCTGTGT-3'	5'- TGAACCCAAGCCACTGGAACA-3'
FAS	5'- TTTTCCTCATGGCTTCACCT-3'	5'- ATGTGGCTGTGCTCATTGAC-3'
BIRC2	5'- ATCGTGCGTCAGAGTGAGC-3'	5'- CGCCGACAAGGAGATACG-3'
BIRC3	5'- GCTTGTCCTTGCTGGTGCAT-3'	5'- TCCCGAGATTAGACTAAGTCCCTT-3'
C-FLAR	5'- TCCATCTTGGGTGCGCCTTC-3'	5'- TCCGGGCCAGTCAACAGAAA-3'
FADD	5'- CCGAGCTCAAGTTCCTATGC-3'	5'- AGGTCTAGGCCGCTCTGC-3'
FAIM2	5'- ATCGGAGCGAAGCAGAGAGG-3'	5'- CACAGTGGTTGAGCATGGGC-3'
FASLG	5'- GAGTCTACCAGCCAGATGCACA-3'	5'- AGGCATGGACCTTGAGTTGG-3'
TNFRSF10A	5'- TACGCCCTGGAGTGACATCG-3'	5'- GACCCAAGCGCCAGAAACAC-3'
TNFRSF10C	5'- CCCTAAAGTTCGTCGTCGTC-3'	5'- TGGTGGCAGAGTAAGCTAGGA-3'
TNF	5'- TGCACTTTGGAGTGATCGGC-3'	5'- TTGTCACTCGGGGTTCGAGA-3'
TNFAIP3	5'- TGCACACTGTGTTTCATCGAG-3'	5'- ACGCTGTGGGACTGACTTTC-3'
TNFRSF10D	5'- AAGTTCGTCGTCTTCATCGTC-3'	5'- GATGGTGGCAGAGTCAACC-3'
TNFRSF1B	5'- CCAGTGCGTTGGACAGAAGG-3'	5'- ATGGCCACCAGGGGAAGAAT-3'
TNFRSF10	5'- TCACAGTGCTCCTGCAGTCT-3'	5'- GCCACTTTTGGAGTACTTGTCC-3'
TRADD	5'- TAGTGCAGCAGGAGGTGAGATG-3'	5'- CCAGCGAGGACTCCACAAAC-3'
TRAF2	5'- GCATACCCGCCATCTTCTC-3'	5'- CGCCGTTCAGGTAGATACG-3'
TRAF5	5'- ATGCCACTTCCCTACTGCTC-3'	5'- CATGGCCCATACTCATCCTT-3'
